# The Effectiveness of Cervical Spine and Diaphragm Manual Therapy Combined with Breathing Re-Education Exercises on Musculoskeletal, Respiratory and Psychophysiological Outcomes in Patients with Non-Specific Chronic Neck Pain: A Randomized Controlled Trial

**DOI:** 10.3390/jcm15114266

**Published:** 2026-05-31

**Authors:** Petros I. Tatsios, Eirini Grammatopoulou, Zacharias Dimitriadis, George A. Koumantakis

**Affiliations:** 1 Physiotherapy Department, School of Health & Care Sciences, University of West Attica (UNIWA), 12243 Athens, Greece; igrammat@uniwa.gr; 2 Laboratory of Advanced Physiotherapy, Physiotherapy Department, School of Health & Care Sciences, University of West Attica (UNIWA), 12243 Athens, Greece; 3 Health Assessment & Quality of Life Laboratory, Physiotherapy Department, University of Thessaly, 35100 Lamia, Greece; zdimitriadis@uth.gr

**Keywords:** chronic neck pain, diaphragm, manual therapy, respiratory dysfunction, breathing re-education exercise

## Abstract

**Background/Objectives:** Dysfunctional breathing interacts with common impairments in patients with non-specific chronic neck pain. This study aimed to assess the effect of combining manual therapy with breathing re-education. **Methods:** A randomized controlled trial with concealed allocation and intention-to-treat analysis, including ninety patients with non-specific chronic neck pain, was employed. Participants were allocated to undertake 10 sessions of either cervical spine and diaphragmatic MT (specifically, Mulligan concept techniques and diaphragmatic doming/release) combined with breathing re-education (experimental group 1, EG1) or cervical spine MT alongside sham diaphragmatic MT (experimental group 2, EG2) or conventional physiotherapy (control group—CG). Interventions lasted 1 month. Primary outcomes were the pain intensity assessed on a 0-to-10 numeric rating scale and the Neck Disability Index percentage. Data were collected at baseline, 1 month, and 4 months post randomization. **Results:** Pain intensity improved more in the EG1 compared to the CG (mean difference −2.15, 95% CI −2.50 to −1.79) and to a lesser extent relative to the EG2 (mean difference −0.42, 95% CI −0.78 to −0.07) at 1 month. Neck disability equally improved in the EG1 (mean difference −14.72, 95% CI −17.55 to −11.89) and the EG2 (mean difference −13.06, 95% CI −15.93 to −10.19) compared to the CG at 1 month. All significant differences for pain and disability noted at one month remained significant at 4 months. EG1 significantly improved in all respiratory-related secondary outcomes compared to EG2 and the CG, both at 1 and 4 months. **Conclusions:** Combining diaphragm manual therapy with breathing re-education led to superior improvements in pain and dysfunctional breathing-related outcomes. **Trial Registration:** NCT05229393.

## 1. Introduction

Neck pain is one of the most common musculoskeletal disorders globally and ranks as the fourth leading cause of years lived with disability [1,2]. Despite this substantial burden, its age-standardized point prevalence, annual incidence, and years lived with disability remained largely unchanged from 1990 to 2017 [1].

The pathophysiology of chronic neck pain (CNP) remains incompletely elucidated and may involve the zygapophyseal joints, intervertebral discs, neural structures, or adjacent soft tissues [3]. Non-specific chronic neck pain (NSCNP) is a complex biopsychosocial disorder presenting with musculoskeletal, respiratory, psychological, and behavioral impairments [4,5]. Dysfunctional breathing (DB), defined as “an alteration in normal breathing patterns that results in dyspnea or other respiratory or non-respiratory chronic symptoms” [6], includes several phenotypes such as hyperventilation syndrome, thoracic-dominant breathing, sporadic deep sighing, forced abdominal expiration, and thoracoabdominal asynchrony [7]. Patients with NSCNP consistently demonstrate abnormal breathing patterns characterized by reduced lung volumes, attenuated chest wall mobility, and diminished respiratory muscle activity [8]. Factors potentially contributing to DB in NSCNP have been detailed in the protocol of the current study [9].

According to the Ontario Protocol for Traffic Injury Management (OPTIMa) Collaboration guidelines, individuals with Grade I–II neck pain lasting more than three months may benefit from a multifaceted treatment approach [4]. Over recent years, growing interest has emerged in manual therapy (MT) and breathing exercises for managing CNP and related musculoskeletal dysfunctions [9,10,11]. Targeted respiratory interventions, including breathing re-education and specialized respiratory exercises, have shown evidence of enhancing therapeutic outcomes [10,11]. Mulligan’s MT techniques have been demonstrated as safe and effective for improving musculoskeletal outcomes in patients with NSCNP [12]. A recent systematic review concluded that diaphragm MT enhances thoracoabdominal expansion, diaphragmatic mobility, and posterior chain flexibility while reducing spinal pain [13].

The rationale for integrating these modalities derives from their synergistic physiological and biomechanical effects. Diaphragmatic breathing physiologically stimulates the vagus nerve, activating parasympathetic tone, reducing sympathetic activity and peripheral inflammatory cytokine production, and enhancing endogenous pain regulation [11]. Relaxation of the diaphragmatic dome combined with respiratory re-education improves diaphragmatic descent and rib cage movement, reducing hypertonicity in the accessory inspiratory muscles (upper trapezius, sternocleidomastoid, anterior scalenes), thereby mitigating mechanical stress on the cervical spine, enhancing mobility, and assisting in correcting forward head posture [14]. Despite this emerging evidence, a substantial research gap persists regarding the combined effects of specific diaphragmatic manual procedures with breathing re-education on musculoskeletal and respiratory outcomes in patients with NSCNP.

The aim of this study was to evaluate the effects of combined cervical and diaphragm MT plus breathing re-education exercises, compared to cervical MT plus sham diaphragmatic techniques and to conventional physiotherapy, on musculoskeletal and respiratory parameters. A randomized controlled trial was designed to test the hypothesis that this combined intervention would yield superior benefits across musculoskeletal, respiratory, and psychophysiological outcomes in patients with NSCNP.

## 2. Materials and Methods

### 2.1. Sample Size Calculation

A convenience sample of ninety patients was recruited on a voluntary basis for this study conducted between June 2022 and July 2023. The G*Power program version 3.1.9.7 was used for sample size calculation [15], performed for a repeated measures ANOVA (within-between interaction) with a medium effect size f = 0.25, a power of 0.95, and an alpha (α) error level of 0.05. The minimum sample required was calculated at 54 patients. The sample size was augmented to accommodate a 10% dropout rate and to comply with the central limit theorem, which posits that a minimum of 30 participants per group is necessary for the sample to exhibit properties of a normal distribution.

### 2.2. Sample Characteristics

The inclusion criteria for the study were as follows: participants’ age range between 25 and 65 years, of either sex, and experiencing chronic mechanical neck pain (duration > 3 months) classified as Grade I or II by the Task Force on Neck Pain [16]. Additionally, participants were required to be fluent in Greek, free from other serious pathologies, demonstrate negative results on specific orthopedic tests (foraminal compression, traction, upper limb tension, and shoulder abduction tests), and exhibit signs of dysfunctional breathing in at least one of the administered biomechanical, biochemical, or psychophysiological assessments.

Individuals were excluded if they presented with any of the following: a history of whiplash, cervical spine trauma or surgery; degenerative spinal disease or cervical disc herniation; radiating pain to the upper extremities; or recent physical therapy targeting the cervical region within the last three months. Further exclusion criteria encompassed pregnancy, systemic illnesses, respiratory or cardiac insufficiency, osteoporosis or metabolic bone diseases, infectious or neoplastic conditions, psychological disorders, and fibromyalgia. Patients exhibiting any clinical “red flags” (such as severe muscle spasms, night pain, unexplained weight loss, daily headaches, dyspnea, confusion, or loss of consciousness) or those who had recently taken muscle relaxants, anti-inflammatories, or analgesics were also excluded.

### 2.3. Ethics

Approval for this study was provided by the Ethics Committee of the University of West Attica, Greece (approval number 51758, 1 June 2022). The research adhered to the stipulations of the Declaration of Helsinki.

### 2.4. Study Design

This investigation was a single-blind randomized controlled trial (RCT) involving a single-blinded assessor, aimed at evaluating the hypothesis that the integration of breathing re-education exercises with cervical spine and diaphragm MT would surpass the efficacy of cervical spine MT or conventional physiotherapy in patients with NSCNP. The reporting of this trial adheres to the Consolidated Standards of Reporting Trials (CONSORT) guidelines [17].

### 2.5. Procedures

A preceding publication [18] also details the procedures followed in this study. Specifically, participants provided a detailed medical history and underwent a series of orthopedic special tests. An assessor with over 20 years’ clinical experience as a physical therapist (A.T.) performed all preliminary assessments relating to the inclusion and exclusion criteria. The assessor also collected all outcomes at three time points: before the intervention, one month immediately after its completion, and 3 months post-intervention.

After completing all preliminary evaluations, participants were randomly assigned to one of three intervention groups using a computer-generated random number sequence through block randomization with specialized software (http://www.randomizer.org/, accessed on 10 May 2022), employing blocks of three participants. A study manager, independent of recruiting, assessment, or intervention delivery, implemented the randomization and concealed allocation procedures. Participants were covertly assigned utilizing sealed, sequentially numbered opaque envelopes, handed to them on the day of their first treatment appointment. The researcher responsible for administering the treatment programs opened each participant’s envelope on the first day of their intervention.

### 2.6. Interventions

The full details of all interventions administered in this study are outlined in the study’s published protocol [9]. A brief summary of their content is provided below. The manual therapy program was delivered by a physical therapist (P.T.), a clinical instructor of manual therapy, with an Orthopedic Manual Therapy (OMT) diploma from a recognized IFOMPT program, certified as Mulligan Practitioner, and 23 years of experience in clinical practice.

#### 2.6.1. The Cervical Spine and Diaphragm ΜΤ Plus Breathing Exercises Group (Experimental Group 1—EG1)

Cervical spine MT was delivered, utilizing inter-vertebral mobilization techniques of the cervical spine according to the Mulligan Concept (Natural Apophyseal Glides—NAGs, Sustained Natural Apophyseal Glides—SNAGs, and traction techniques) [19], in conjunction with cervicothoracic muscles stretching techniques, as previously described in detail [9]. Subsequently, diaphragmatic MT was implemented, incorporating the doming diaphragmatic technique and diaphragmatic release technique [20], followed by breathing re-education exercises [21].

#### 2.6.2. The Cervical Spine Plus Sham Diaphragmatic MΤ Group (Experimental Group 2—EG2)

Cervical spine MT was delivered, following the same approach as utilized for EG1. Participants additionally received sham diaphragmatic MT utilizing a therapeutic ultrasound device (Sonopuls 692 Enraf-Nonius, Rotterdam, The Netherlands) for 10 min while sitting, with the device running but set to 0 W/cm^2^ intensity level, thereby simulating treatment without delivering any genuine therapeutic effect [22]. The same therapist consistently administered the sham ultrasound. Furthermore, verbal guidance on incorporating postural adjustments throughout everyday tasks (i.e., cleaning, sweeping, sitting, and driving) was administered.

#### 2.6.3. The Conventional Physiotherapy Group (Control Group—CG)

Participants assigned to the Control Group (CG) underwent a standard conventional physiotherapy regimen. The session commenced with the application of Transcutaneous Electrical Nerve Stimulation (TENS) for 15 min, utilizing a frequency of 80 Hz and a pulse width of 250 μs, with electrodes placed bilaterally over the trapezius and suboccipital areas. This was followed by a 10 min session of pulsed microwave diathermy (Enraf-Nonius RADARMED 950+) administered with the patient in a seated position. Finally, patients received 15 min of slow-paced, deep soft-tissue massage. These manual techniques, primarily gliding and kneading, specifically targeted the upper, middle, and lower fibers of the trapezius, as well as the levator scapulae and splenius capitis muscles [23].

### 2.7. Outcomes Measures

Primary outcomes were the Pain Intensity Numeric Rating Scale (PI-NRS) and Neck Disability Index (NDI). Secondary outcomes were the Tampa Scale for Kinesiophobia—Greek version (TSK-GR), Hospital Anxiety and Depression Scale—Greek version (HADS-GR), Nijmegen Questionnaire (NQ), Hi-Lo test, Breath Holding Time (BHT) test, Single Breath Count (SBC) test, Capnography—End Tidal CO_2_, Respiratory Rate (RR), and Chest Wall Expansion (CWE). All outcome measures are described in detail in the Supplementary Materials.

### 2.8. Statistical Analysis

The Shapiro–Wilk test with complementary evaluation of the distribution curves and the Q-Q plots was used to analyze all outcomes per group for normality of distribution. Baseline comparisons between groups were conducted using one-way ANOVA and chi-square tests.

The primary analysis involved a 3 × 3 two-way mixed ANOVA, which included a repeated-measures factor (time) and a between-groups factor (group) for each outcome. Mauchly’s test assessed the sphericity assumption. Partial eta squared (η^2^p) effect size was utilized to assess the proportion of variance attributable to treatment differences. The power achieved for each outcome was also reported. Bonferroni-corrected post hoc univariate tests were conducted to analyze differences within groups across time points and to assess differences between groups at each time point.

To complement this analysis, ANCOVA was also used, as in interventional experimental designs it removes extraneous influences such as pre-test differences, so that the actual between-group differences could then be compared with the calculated within-group differences. Specifically, two separate ANCOVAs between the three groups were employed per continuous outcome, using baseline values as a covariate, to calculate the [1-month–Baseline] and the [4-month–Baseline] differences. Univariate post hoc ANOVAs were then used to examine within-group and between-group differences. To reduce the risk of type I error, *p*-values were adjusted using the Bonferroni correction method for multiple comparisons. Statistical significance was defined as *p* < 0.05.

For the categorical outcome of the Hi-Lo Test, Pearson’s chi-square test was applied at each time point to identify between-group differences.

The “intention-to-treat” (ITT) principle was used for all analyses, ensuring that all subjects randomly assigned to the study groups were analyzed according to their assigned groups. To preserve this principle and retain all randomized participants in the analysis, missing outcome values were replaced with the observed mean of the participant’s allocated treatment group, an approach consistent with simple single-imputation methods that assume data are missing completely at random (MCAR) [24]. The known limitations of this approach, and the more robust alternatives recommended in contemporary guidance [24], are addressed in the Discussion. A complete-case sensitivity analysis was performed to confirm the robustness of effect estimates. Data were analyzed using IBM SPSS version 29.0.2.0.

## 3. Results

### 3.1. Flow of Participants Through the Study

**Figure 1:**
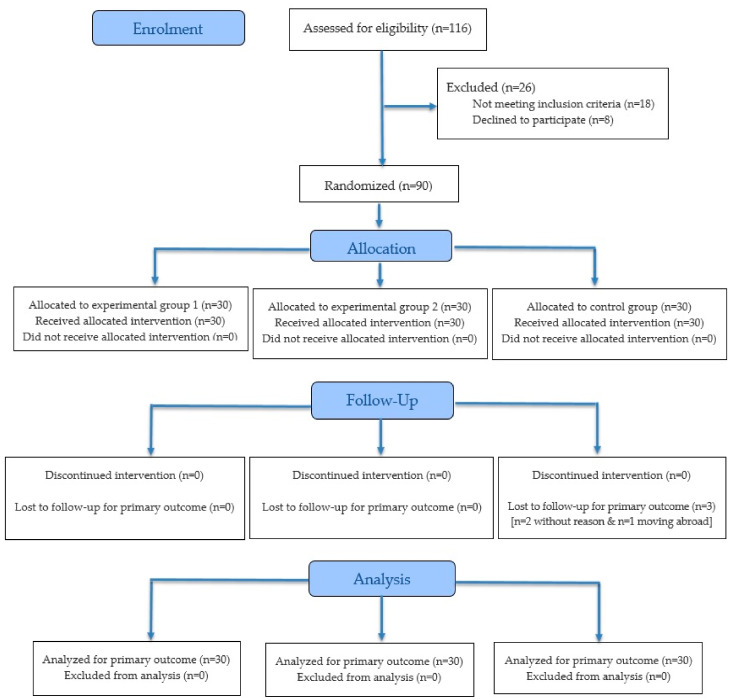
Flow of participants through the trial.

Screening was conducted on 116 patients between June 2022 and August 2023 for eligibility. A total of 90 participants were eligible and were randomly allocated to the EG1 (n = 30), the EG2 (n = 30) and the CG (n = 30). A detailed flowchart of participant allocation and analysis is presented in Figure 1.

### 3.2. Demographics

A total of 90 patients (54 women) diagnosed with non-specific chronic neck pain (NSCNP) participated in this study. The mean age of the participants was 41.25 years (SD = 10.93). All patients had been referred for physiotherapy at a private practice and experienced a pain episode lasting longer than three months, with an average duration of 6.39 (SD = 1.72) months. The demographic and clinical baseline characteristics of the participants, categorized by their assigned intervention group, are presented in Table 1. Statistical analysis revealed no significant baseline differences among the groups regarding age, sex distribution, anthropometric measurements, or symptom duration, confirming that the randomization process was successful and the groups were comparable prior to the interventions. Only three volunteers, all from the control group, withdrew after completing all treatments. Two of them did so without providing an explanation, while one relocated abroad.

### 3.3. Outcomes

**Figure 2:**
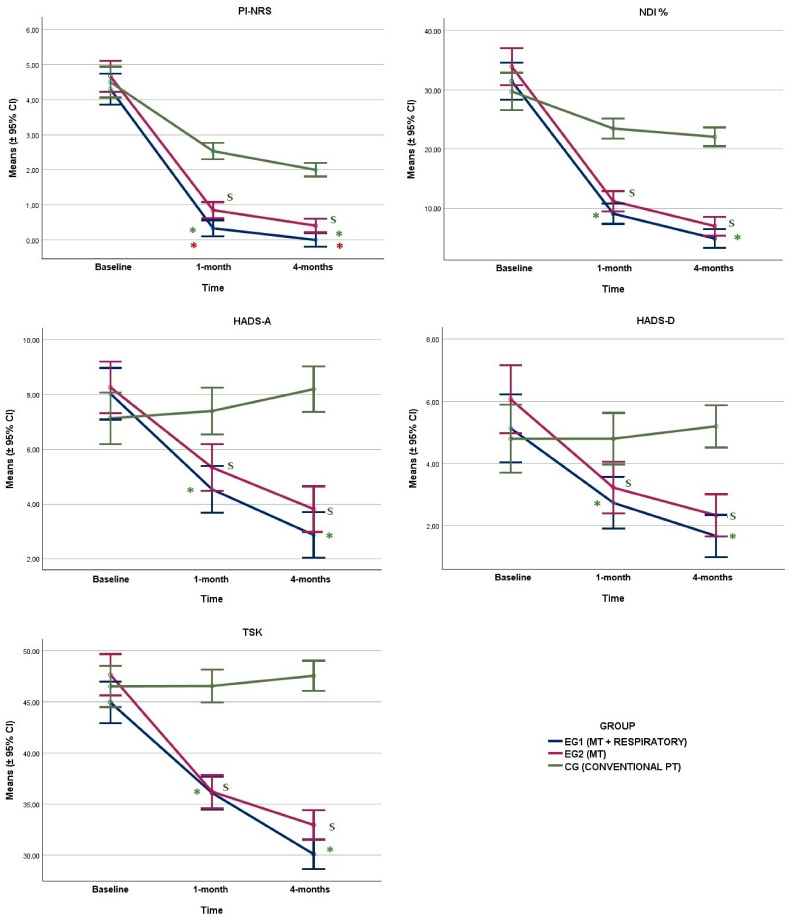
Change over time of the musculoskeletal-based outcomes (primary and secondary) for the three groups of participants. The post hoc pairwise comparisons from the mixed ANOVA analysis, with *p*-values adjusted with the Bonferroni correction method, revealed significant differences between EG1 and EG2 (red asterisks), EG1 and CG (green asterisks), and EG2 and CG (S sign) noted at each time point. * *p* < 0.05.

**Figure 3:**
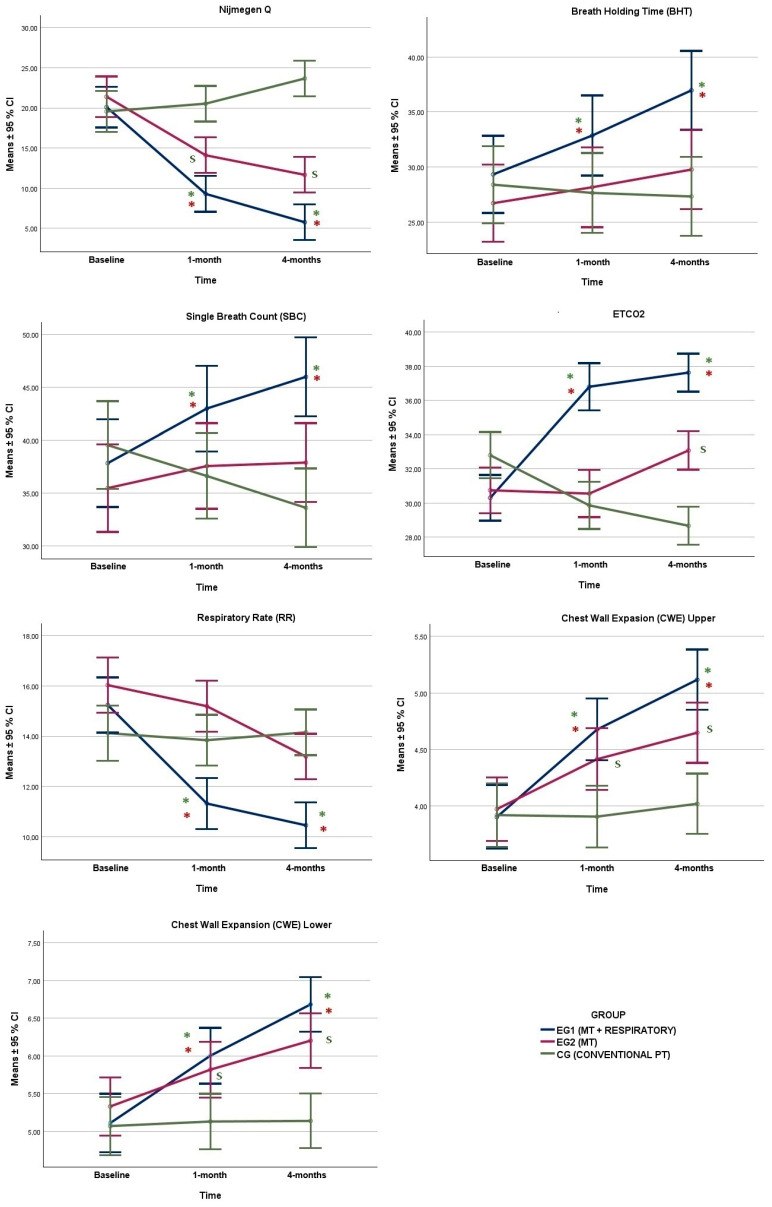
Change over time of the respiratory-based outcomes (secondary) for the three groups of participants. The post hoc pairwise comparisons from the mixed ANOVA analysis, with *p*-values adjusted with the Bonferroni correction method, revealed significant differences between EG1 and EG2 (red asterisks), EG1 and CG (green asterisks), and EG2 and CG (S sign) noted at each time point. * *p* < 0.05.

Overall, our findings indicated that the combined experimental intervention (EG1) yielded the most significant improvements across one of the two primary outcomes (pain intensity) and all the secondary respiratory outcomes compared to the other groups. All outcomes were normally distributed, therefore considered amenable to parametric analysis. The mixed ANOVA indicated that all outcomes showed significant improvement over time (*p* < 0.001), particularly for EG1 and EG2 between the baseline and the 1-month post-randomization time point. Those improvements were maintained for EG1 and EG2 at 4-months post-randomization (Table 2, Figure 2 and Figure 3). The interaction effect (time x group) was significant (*p* < 0.001), with a large effect size (η^2^p > 0.14) and high power for all outcomes (Table 1). Post hoc pairwise comparisons (Bonferroni corrected) at each time point identified significant differences between EG1 and EG2 for pain (Figure 2) and all respiratory-related outcomes (Figure 3).

Via the ANCOVA analyses, between-group differences (95% CIs) were calculated using Bonferroni-corrected post hoc pairwise comparisons. PI-NRS on a 0–10 scale improved more in EG1 compared with CG (MD −2.15, 95% CI −2.50 to −1.79) and to a lesser extent relative to EG2 (MD −0.42, 95% CI −0.78 to −0.07) at the 1-month follow-up. Disability on NDI% improved equally in EG1 (MD −14.72, 95% CI −17.55 to −11.89) and EG2 (MD −13.06, 95% CI −15.93 to −10.19) compared with CG at the 1-month follow-up. For TSK, similar improvements were noted for EG1 (MD −9.86, 95% CI −12.35 to −7.37) and EG2 (MD −10.78, 95% CI −13.27 to −8.30) compared with CG at the 1-month follow-up. For HADS-A, similar improvements were noted for EG1 (MD −3.37, 95% CI −4.54 to −2.20) and EG2 (MD −2.70, 95% CI −3.88 to −1.52) compared with CG at the 1-month follow-up. For HADS-D, nearly parallel improvements were noted for EG1 (MD −2.22, 95% CI −3.38 to −1.06) and EG2 (MD −2.16, 95% CI −3.34 to −0.98) compared with CG at the 1-month follow-up. All differences for PI-NRS, NDI%, TSK, HADS-A and HADS-D noted at the 1-month follow-up remained significant at the 4-month follow-up. EG1 registered significant improvements in all other respiratory-related secondary outcomes compared with EG2 and CG, both at the 1-month follow-up and at the 4-month follow-up (Table 2, Figure 3).

For the Hi-Lo Test, Pearson’s chi-square applied at each time point identified no significant between-group differences at baseline. In EG1, 25/30 patients initially presented with a Hi breathing pattern. Significant between-group differences emerged at the 1-month and at the 4-month follow up, as the breathing pattern of nearly all EG1 patients changed to Lo from Hi (28/30 patients at 1 month and 27/30 patients at 4 months), as shown in Table 3.

### 3.4. Adverse Events Monitoring

No adverse events were reported for any of the participants throughout the trial period.

## 4. Discussion

This study demonstrates that combining cervical spine and diaphragm MT with breathing re-education exercises (EG1) produced significantly greater improvements in pain intensity—both short- and long-term—and across all three dimensions of respiratory dysfunction compared with cervical MT plus sham diaphragmatic MT (EG2). Both experimental groups showed superior outcomes versus conventional physiotherapy (CG) for nearly all variables, with effects maintained at 3 months post-treatment.

Several studies have established connections between breathing dysfunction and functional limitations in patients with CNP. Our prior systematic review with meta-analysis [25] revealed limited evidence on the effectiveness of spinal/diaphragmatic MT, trunk stabilization exercises, or respiratory exercises in significantly improving pain, disability, and respiratory outcomes immediately post-treatment. The current study addresses these gaps through its combined intervention design and comprehensive assessment across biochemical (ETCO_2_, BHT, SBC), biomechanical (Hi-Lo pattern, RR, CWE), and psychophysiological (NQ, HADS, TSK) dimensions of dysfunctional breathing [7,26].

Diaphragmatic MT and breathing exercises specifically aim to release the diaphragmatic dome, enhance caudal excursion, increase rib cage mobility, and reduce excessive tension in accessory inspiratory muscles, thereby improving all three DB dimensions [8]. Our systematic review identified only one prior RCT [27] that combined diaphragm MT with cervical articular MT in CNP patients; their pain findings differed from ours, possibly because the three-session duration was insufficient for clinically meaningful effects, though their disability findings aligned. A more recent RCT [28] incorporating once-weekly diaphragm myofascial release into a four-week exercise program supported our disability findings, with significant within-group improvements and notable CWE changes favoring the intervention group. McLaughlin [29] proposed that while 5–6 sessions of breathing retraining over approximately one month may suffice for many patients, complex cases may require longer durations.

Pain intensity improved significantly more in EG1 than EG2 and CG at both time points, consistent with broader literature supporting multimodal physiotherapy for CNP [30,31,32,33,34,35], though contrasting with two studies that found no between-group differences for pain [33,34]. Heterogeneity in treatment protocols and durations (2–8 weeks) across prior studies limits direct comparison. Our disability findings align with studies reporting only within-group improvements [35], suggesting that cervical MT alone may suffice for short-term disability reduction. For chest wall expansion, significant within-group improvements in both upper and lower CWE in EG1 and EG2 likely reflect compensatory rib cage mobility during maximal inspiration—a response not necessarily predicted by quiet-breathing diaphragmatic retraining. Our findings are in partial agreement with Sakuna et al. [36], who demonstrated that breathing retraining combined with chest wall mobilization in patients with CNP led to a trend towards increased CWE, while they contrast those of Mohan et al. [31], who found no significant CWE change, possibly due to differences in exercise modality and frequency.

Psychological outcomes (TSK, HADS-A, HADS-D) showed statistically significant improvements in both experimental groups versus CG, but no significant difference between EG1 and EG2, consistent with previous RCTs [30,33]. Another study [32], using different measurement tools (DASS21), reported significant stress reductions following an 8-week breathing/relaxation program; however, methodological differences limit direct comparison.

Mulligan’s MT techniques activate multisystem mechanisms spanning biomechanical, neurophysiological, and non-specific pathways [37]. A recent systematic review found SNAGs combined with other interventions superior to exercise or muscle-energy techniques for pain and disability in CNP, though with very low certainty evidence; notably, none of the included studies assessed respiratory outcomes [12]. The present study extends this evidence base by demonstrating that incorporating diaphragmatic MT and breathing re-education produces additional benefits for dysfunctional breathing parameters (ETCO_2_, NQ, BHT, SBC, Hi-Lo pattern)—outcomes that have not been assessed in any previous RCT of this combination.

In a case series of 29 outpatients with neck or back pain who had reached a plateau with conventional MT and education, McLaughlin et al. [38] reported a 7 mmHg improvement in ETCO_2_ following individualized breathing retraining supplemented with MT—consistent with our 5 mmHg improvement in EG1. The improved Hi-Lo breathing pattern observed exclusively in EG1 likely reflects diaphragmatic release techniques combined with breathing re-education, evidenced by enhanced diaphragmatic function and reduced accessory respiratory muscle tension. The observed pain reduction in EG1 may be associated with slowing the breathing rate to approximately 4–6 breaths/min, a pace that synchronizes blood pressure and heart rate oscillations and enhances parasympathetic function [39]. The biochemical dimension was also normalized in EG1 (ETCO_2_ > 35 mmHg, BHT > 33 s, SBC > 43 counts, RR < 10 breaths/min), accompanied by psychophysiological improvements (TSK < 36, HADS-A < 5, HADS-D < 3, NQ < 10).

CNP is heavily influenced by psychological factors [40], and similar increases in respiratory dysfunction have been observed in individuals with poor mental health [5]. Because breathing exercises are effective in reducing anxiety and stress [11], the observed pain and disability improvements may partly reflect this mechanism. Pain reduction in EG1 may further reflect central analgesic effects: slow diaphragmatic breathing stimulates the vagus nerve, reducing sympathetic tone, peripheral cytokine production, and pain-related cortical activation, while modulating endogenous opioid effects [39]. The integrated approach (EG1) produced clinically significant improvements in pain and disability versus CG; however, differences between EG1 and EG2 for these primary outcomes did not reach the established MDC/MCID thresholds. Statistically significant differences favoring EG1 over both other groups were observed for pain and all respiratory secondary outcomes, suggesting that respiratory interventions may serve as beneficial adjuncts in NSCNP management.

Several methodological features supported the internal validity of this RCT: comparable group creation via allocation concealment to prevent selection bias, minimized attrition through close communication, strict intervention adherence preventing group contamination, reliable and valid measurement instruments, assessor-blinded outcome assessment, and clearly defined interventions.

Only three participants (3.3%) were lost to follow-up, all from the control group, and missing values were replaced with the observed mean of the participant’s allocated treatment group. Although the proportion of missing data was small, the choice of imputation method warrants critical reflection. Single mean imputation has well-recognized methodological limitations: it artificially reduces between-subject variance, attenuates standard errors, inflates the type I error rate, and depends on the strong and rarely justified assumption that data are missing completely at random (MCAR)—that is, that the probability of missingness is unrelated to either observed or unobserved values [24]. In clinical trials, the MCAR assumption is seldom plausible, since the reasons participants drop out are often related to their treatment response, baseline severity, or unmeasured factors. The fact that all three dropouts occurred in the control group is itself suggestive of an informative pattern of missingness rather than a purely random one.

According to contemporary recommendations [24], a more methodologically robust primary analytical strategy under the more plausible missing-at-random (MAR) assumption would have been a mixed-effects model for repeated measures (MMRM). MMRM yields unbiased estimates of treatment effects without requiring any imputation, uses all available observations from each randomized participant, models the within-subject correlation structure of repeated measurements directly, and accommodates unbalanced data across time points [24]. As a likelihood-based method, MMRM also handles MAR mechanisms appropriately without explicit weighting or imputation. Alternatively, multiple imputation (MI) generates several plausible values for each missing observation from an imputation model that includes auxiliary variables, then pools the results across imputed datasets using Rubin’s rules to appropriately propagate the additional uncertainty introduced by imputation [24]. Either approach would have been preferable to single mean imputation as the primary analysis in the present trial. Reassuringly, the complete-case sensitivity analysis we performed confirmed that the direction and statistical significance of treatment effects were maintained, supporting the robustness of our findings despite this methodological limitation. Future RCTs in this domain should adopt MMRM (or MI) as the primary analytical strategy and report at least one alternative method as a pre-specified sensitivity analysis, with the imputation model and missingness assumption transparently reported [24].

Other limitations include: (i) the absence of 12-month follow up, which precludes assessment of long-term durability of treatment effects; (ii) recruitment from a single private physiotherapy practice, limiting generalizability to hospital-based pain clinics, multidisciplinary pain centers, or public healthcare settings; (iii) a relatively mild-to-moderate clinical profile (mean pain 4.5/10, average duration 6.4 months) reflecting stringent inclusion/exclusion criteria, limiting applicability to more severely affected populations; (iv) inability to determine the independent contributions of diaphragmatic MT versus breathing re-education, since both components were delivered in the same session; (v) potential patient fatigue from multiple outcome measures across three time points; and (vi) absence of progressively prescribed respiratory muscle strengthening exercises as a potentially complementary therapeutic component warranting future investigation.

Clinicians managing NSCNP should consider the multifactorial nature of the condition by addressing musculoskeletal, respiratory, and psychophysiological dimensions in both assessment and treatment. Breathing re-education exercises and diaphragmatic release techniques can serve as potentially valuable adjuncts to conventional manual therapy.

## 5. Conclusions

This is the first single-blind RCT to provide evidence regarding the extent of improvement in respiratory and musculoskeletal parameters following a combined intervention of diaphragm treatment, cervical MT, and breathing re-education exercises for patients with NSCNP. The findings further support the notion that targeting distant somatic tissues such as the diaphragm can positively influence a relatively remote painful area such as the cervical spine, owing to their shared innervation and structural continuity through fascial connections.

## Figures and Tables

**Table 1:** Baseline demographic characteristics and pain duration of the participants across the three groups.

	EG1 (n = 30)	EG2 (n = 30)	CG (n = 30)	Statistic	*p*
Sex (Male/Female)	15/15	8/22	13/17	x^2^ = 3.61	0.16
Age (y)	41.20 ± 11.53	39.93 ± 8.00	42.63 ± 12.87	F = 0.45	0.64
Height (m)	171.93 ± 7.53	171.20 ± 6.24	172.00 ± 9.85	F = 0.09	0.91
Body Mass (kg)	74.50 ± 18.01	73.97 ± 14.94	77.30 ± 13.99	F = 0.39	0.68
Body Mass Index (kg/m^2^)	24.96 ± 4.63	25.11 ± 4.07	26.10 ± 4.18	F = 0.62	0.54
Pain duration (months)	6.47 ± 1.57	6.43 ± 1.89	6.27 ± 1.72	F = 0.11	0.89

y: years, kg: kilograms, m: meters.

**Table 2:** Descriptive statistics (mean ± SD) and interaction effect (time x group) of the main and the secondary outcomes for all measurement time points per treatment group.

	EG1 (n = 30)	EG2 (n = 30)	CG (n = 30)	F	*p*	η^2^p	Power
Main Outcomes							
PI-NRS (0–10)							
Baseline	4.30 ± 1.18	4.67 ± 1.32	4.50 ± 1.14	22.16	<0.001	0.34	1.00
1 month	0.33 ± 0.48 ^a,b^	0.85 ± 0.63	2.53 ± 0.78				
4 months	0.00 ± 0.00 ^a,b^	0.41 ± 0.54	2.00 ± 0.74				
NDI (0–100)							
Baseline	31.47 ± 7.81	33.93 ± 11.48	29.73 ± 5.86	38.90	<0.001	0.47	1.00
1 month	9.07 ± 4.02 ^b^	11.18 ± 4.49 ^b^	23.47 ± 5.53				
4 months	4.87 ± 2.71 ^b^	6.96 ± 4.21 ^b^	22.07 ± 5.72				
Secondary outcomes							
TSK							
Baseline	44.97 ± 6.23	47.67 ± 5.15	46.53 ± 5.28	56.95	<0.001	0.57	1.00
1 month	36.10 ± 4.91 ^b^	36.22 ± 3.78 ^b^	46.57 ± 4.62				
4 months	30.10 ± 3.72 ^b^	32.96 ± 4.05 ^b^	47.57 ± 4.34				
HADS-A							
Baseline	8.03 ± 2.09	8.27 ± 3.29	7.13 ± 2.27	33.19	<0.001	0.43	1.00
1 month	4.53 ± 2.05 ^b^	5.33 ± 2.13 ^b^	7.40 ± 2.77				
4 months	2.87 ± 1.85 ^b^	3.81 ± 2.35 ^b^	8.2 ± 2.62				
HADS-D							
Baseline	5.13 ± 2.43	6.07 ± 3.80	4.80 ± 2.63	18.00	<0.001	0.29	1.00
1 month	2.73 ± 1.76	3.22 ± 2.49	4.80 ± 2.54				
4 months	1.67 ± 1.09	2.33 ± 1.98	5.20 ± 2.32				
NQ							
Baseline	20.10 ± 5.57	21.40 ± 8.22	19.57 ± 6.91	65.55	<0.001	0.60	1.00
1 month	9.30 ± 3.67 ^a,b^	14.11 ± 6.14 ^b^	20.53 ± 7.82				
4 months	5.77 ± 2.25 ^a,b^	11.67 ± 6.71 ^b^	23.67 ± 7.88				
BHT							
Baseline	29.33 ± 10.96	26.72 ± 8.02	28.40 ± 9.81	22.46	<0.001	0.34	1.00
1 month	32.87 ± 11.91 ^a,b^	28.17 ± 8.02	27.65 ± 9.72				
4 months	36.97 ± 11.74 ^a,b^	29.77 ± 8.56	27.33 ± 9.09				
SBC							
Baseline	37.85 ± 8.65	35.48 ± 11.27	39.55 ± 13.79	20.19	<0.001	0.32	1.00
1 month	43.00 ± 9.42 ^a,b^	37.57 ± 10.92	36.63 ± 12.82				
4 months	46.00 ± 8.61 ^a,b^	37.90 ± 9.89	33.62 ± 12.00				
ETCO_2_							
Baseline	30.31 ± 3.83	30.75 ± 3.69	32.80 ± 3.57	30.86	<0.001	0.41	1.00
1 month	36.80 ± 4.19 ^a,b^	30.56 ± 3.23	29.86 ± 3.93				
4 months	37.63 ± 2.32 ^a,b^	33.08 ± 3.61 ^b^	28.67 ± 3.15				
RR							
Baseline	15.24 ± 2.51	16.03 ± 3.28	14.12 ± 3.17	7.82	<0.001	0.15	1.00
1 month	11.32 ± 1.96 ^a,b^	15.19 ± 2.93	13.84 ± 3.32				
4 months	10.45 ± 2.24 ^a,b^	13.19 ± 2.45	14.15 ± 2.79				
CWE-Up							
Baseline	3.90 ± 0.96	3.97 ± 0.75	3.92 ± 0.55	38.92	<0.001	0.47	1.00
1 month	4.68 ± 0.90 ^a,b^	4.41 ± 0.78 ^b^	3.91 ± 0.53				
4 months	5.12 ± 0.74 ^a,b^	4.65 ± 0.89 ^b^	4.02 ± 0.53				
CWE-Lw							
Baseline	5.11 ± 1.26	5.33 ± 1.14	5.07 ± 0.73	38.47	<0.001	0.47	1.00
1 month	6.00 ± 1.21 ^a,b^	5.82 ± 1.06 ^b^	5.13 ± 0.73				
4 months	6.68 ± 1.12 ^a,b^	6.20 ± 1.11 ^b^	5.14 ± 0.70				

PI-NRS: Pain intensity numeric rating scale, NDI: Neck Disability Index, TSK: Tampa Scale of Kinesiophobia, HADS: Hospital Anxiety and Depression Scale, NQ: Nijmegen Questionnaire, BHT: Breath Holding Time, SBC: Single Breath Count, ETCO_2_: End Tidal CO_2_, RR: Respiratory Rate, and CWE: Chest Wall Expansion, ^a^: significantly different from the EG2, ^b^: significantly different from CG.

**Table 3:** Descriptive statistics and Pearson’s chi-square test of the secondary outcome of breathing pattern (Hi vs. Lo) for each of the measurement time points to identify between-group differences.

	EG1 (n = 30)	EG2 (n = 30)	CG (n = 30)	X^2^	*p*
**Hi/Lo**					
Baseline	25/5	28/2	28/2	2.22	*0.33*
1 month	2/28	25/2	28/2	62.98	*<0.001*
4 months	3/27	22/5	29/1	54.12	*<0.001*

## Data Availability

The data presented in this study are available upon request from the corresponding author. The data are not publicly available due to the applicable data protection law in Greece (Law 4624/2019).

## Supplementary Materials

The following supporting information can be downloaded at: https://www.mdpi.com/article/10.3390/jcm15114266/s1, Outcome Measures and Adverse Events Monitoring. CONSORT 2025 checklist of information to include when reporting a randomised trial.

## Abbreviations

The following abbreviations are used in this manuscript:

EG1	Experimental Group 1
EG2	Experimental Group 2
CG	Control Group
CNP	Chronic Neck Pain
NSCNP	Non-Specific Chronic Neck Pain
PI-NRS	Pain Intensity-Numeric Rating Scale
NDI	Neck Disability Index
HADS-D	Hospital Anxiety and Depression Scale—Depression
HADS-A	Hospital Anxiety and Depression Scale—Anxiety
TSK	Tampa Scale of Kinesiophobia
NQ	Nijmegen Questionnaire
SBC	Single Breath Count
BHT	Breath Holding Time
CWE	Chest Wall Expansion

## References

[1] GBD 2021 Neck Pain Collaborators (2024). Global, regional, and national burden of neck pain, 1990–2020, and projections to 2050: A systematic analysis of the Global Burden of Disease Study 2021. Lancet Rheumatol..

[2] Cieza A., Causey K., Kamenov K., Hanson S.W., Chatterji S., Vos T. (2021). Global estimates of the need for rehabilitation based on the Global Burden of Disease study 2019: A systematic analysis for the Global Burden of Disease Study 2019. Lancet.

[3] Lindenmann S., Tsagkaris C., Farshad M., Widmer J. (2022). Kinematics of the Cervical Spine Under Healthy and Degenerative Conditions: A Systematic Review. Ann. Biomed. Eng..

[4] Côté P., Wong J.J., Sutton D., Shearer H.M., Mior S., Randhawa K., Ameis A., Carroll L.J., Nordin M., Yu H. (2016). Management of neck pain and associated disorders: A clinical practice guideline from the Ontario Protocol for Traffic Injury Management (OPTIMa) Collaboration. Eur. Spine J..

[5] Stephen S., Brandt C., Olivier B. (2022). Neck Pain and Disability: Are They Related to Dysfunctional Breathing and Stress?. Physiother. Can..

[6] Barker N., Everard M.L. (2015). Getting to grips with ‘dysfunctional breathing’. Paediatr. Respir. Rev..

[7] Boulding R., Stacey R., Niven R., Fowler S.J. (2016). Dysfunctional breathing: A review of the literature and proposal for classification. Eur. Respir. Rev..

[8] Borisut S., Tantisuwat A., Gaogasigam C. (2021). The study of respiratory muscles activation during respiratory muscle strength effort in adult females with chronic neck pain. J. Phys. Ther. Sci..

[9] Tatsios P.I., Grammatopoulou E., Dimitriadis Z., Koumantakis G.A. (2022). The Effectiveness of Manual Therapy in the Cervical Spine and Diaphragm, in Combination with Breathing Reeducation Exercises, in Patients with Non-Specific Chronic Neck Pain: Protocol for Development of Outcome Measures and a Randomized Controlled Trial. Diagnostics.

[10] Cefalì A., Santini D., Lopez G., Maselli F., Rossettini G., Crestani M., Lullo G., Young I., Dunning J., de Abreu R.M. (2025). Effects of Breathing Exercises on Neck Pain Management: A Systematic Review with Meta-Analysis. J. Clin. Med..

[11] Jafari H., Gholamrezaei A., Franssen M., Van Oudenhove L., Aziz Q., Van den Bergh O., Vlaeyen J.W.S., Van Diest I. (2020). Can Slow Deep Breathing Reduce Pain? An Experimental Study Exploring Mechanisms. J. Pain.

[12] Barbosa-Silva J., Luc A., Sobral de Oliveira-Souza A.I., Martins de Abreu R., Cipriano J., de Schaetzen M., Pitance L., Armijo-Olivo S. (2025). The Effectiveness of Mulligan’s Techniques in Non-Specific Neck Pain: A Systematic Review and Meta-Analysis. Physiother. Res. Int..

[13] Fernández-López I., Peña-Otero D., Atín-Arratibel M., Eguillor-Mutiloa M. (2021). Effects of Manual Therapy on the Diaphragm in the Musculoskeletal System: A Systematic Review. Arch. Phys. Med. Rehabil..

[14] Hidalgo B., Hall T., Bossert J., Dugeny A., Cagnie B., Pitance L. (2017). The efficacy of manual therapy and exercise for treating non-specific neck pain: A systematic review. J. Back. Musculoskelet. Rehabil..

[15] Faul F., Erdfelder E., Lang A.G., Buchner A. (2007). G*Power 3: A flexible statistical power analysis program for the social, behavioral, and biomedical sciences. Behav. Res. Methods.

[16] Nordin M., Carragee E.J., Hogg-Johnson S., Weiner S.S., Hurwitz E.L., Peloso P.M., Guzman J., van der Velde G., Carroll L.J., Holm L.W. (2008). Assessment of Neck Pain and Its Associated Disorders: Results of the Bone and Joint Decade 2000–2010 Task Force on Neck Pain and Its Associated Disorders. Spine.

[17] Hopewell S., Chan A.W., Collins G.S., Hróbjartsson A., Moher D., Schulz K.F., Tunn R., Aggarwal R., Berkwits M., Berlin J.A. (2025). CONSORT 2025 statement: Updated guideline for reporting randomized trials. Nat. Med..

[18] Tatsios P.I., Grammatopoulou E., Dimitriadis Z., Koumantakis G.A. (2025). The Efectiveness of Manual Therapy in the Cervical Spine and Diaphragm, in Combination with Breathing Re-Education Exercises, on the Range of Motion and Forward Head Posture in Patients with NonSpecific Chronic Neck Pain: A Randomized Controlled Trial. Healthcare.

[19] Hing W., Hall T., Mulligan B. (2019). The Mulligan Concept of Manual Therapy.

[20] Chaitow L. (2004). Breathing pattern disorders, motor control, and low back pain. J. Osteopath. Med..

[21] Laborde S., Iskra M., Zammit N., Borges U., You M., Sevoz-Couche C., Dosseville F. (2021). Slow-paced breathing: Influence of inhalation/exhalation ratio and of respiratory pauses on cardiac vagal activity. Sustainability.

[22] Valenza M.C., Cabrera-Martos I., Torres-Sánchez I., Garcés-García A., Mateos-Toset S., Valenza-Demet G. (2015). The Effects of Doming of the Diaphragm in Subjects with Short-Hamstring Syndrome: A Randomized Controlled Trial. J. Sport Rehabil..

[23] Rodríguez-Huguet M., Rodríguez-Almagro D., Rodríguez-Huguet P., Martín-Valero R., Lomas-Vega R. (2020). Treatment of Neck Pain with Myofascial Therapies: A Single Blind Randomized Controlled Trial. J. Manip. Physiol. Ther..

[24] Dziura J.D., Post L.A., Zhao Q., Fu Z., Peduzzi P. (2013). Strategies for dealing with missing data in clinical trials: From design to analysis. Yale J. Biol. Med..

[25] Tatsios P.I., Grammatopoulou E., Dimitriadis Z., Papandreou M., Paraskevopoulos E., Spanos S., Karakasidou P., Koumantakis G.A. (2022). The Effectiveness of Spinal, Diaphragmatic, and Specific Stabilization Exercise Manual Therapy and Respiratory-Related Interventions in Patients with Chronic Nonspecific Neck Pain: Systematic Review and Meta-Analysis. Diagnostics.

[26] Vidotto L.S., Carvalho C.R.F., Harvey A., Jones M. (2019). Dysfunctional breathing: What do we know?. J. Bras. Pneumol..

[27] Simoni G., Bozzolan M., Bonnini S., Grassi A., Zucchini A., Mazzanti C., Oliva D., Caterino F., Gallo A., Da Roit M. (2021). Effectiveness of standard cervical physiotherapy plus diaphragm manual therapy on pain in patients with chronic neck pain: A randomized controlled trial. J. Bodyw. Mov. Ther..

[28] Haghighat F., Moradi R., Rezaie M., Yarahmadi N., Ghaffarnejad F. (2025). Added Value of Diaphragm Myofascial Release on Forward Head Posture and Chest Expansion in Patients with Neck Pain: A Randomized Controlled Trial. J. Bodyw. Mov. Ther..

[29] McLaughlin L. (2009). Breathing evaluation and retraining in manual therapy. J. Bodyw. Mov. Ther..

[30] Beltran-Alacreu H., López-de-Uralde-Villanueva I., Fernández-Carnero J., La Touche R. (2015). Manual therapy, therapeutic patient education, and therapeutic exercise, an effective multimodal treatment of nonspecific chronic neck pain: A randomized controlled trial. Am. J. Phys. Med. Rehabil..

[31] Mohan V., Ahmad N.B., Tambi N.B. (2016). Effect of respiratory exercises on neck pain patients: A pilot study. Pol. Ann. Med..

[32] Metikaridis D., Hadjipavlou A., Artemiadis A., Chrousos G., Darviri C. (2017). Effect of a stress management program on subjects with neck pain: A pilot randomized controlled trial. J. Back Musculoskelet. Rehabil..

[33] Kaya D.Ö., Çelenay Ş.T. (2019). Effectiveness of relaxation training in addition to stabilization exercises in chronic neck pain: A randomized clinical trial. Turk. J. Physiother. Rehabil..

[34] Dareh-Deh H.R., Hadadnezhad M., Letafatkar A., Peolsson A. (2022). Therapeutic routine with respiratory exercises improves posture, muscle activity, and respiratory pattern of patients with neck pain: A randomized controlled trial. Sci. Rep..

[35] Anwar S., Arsalan A., Zafar H., Ahmad A., Hanif A. (2022). Effects of breathing reeducation on cervical and pulmonary outcomes in patients with non specific chronic neck pain: A double blind randomized controlled trial. PLoS ONE.

[36] Sakuna M., Mekhora K., Jalajondeja W., Jalajondeja C. (2020). Breathing retraining with chest wall mobilization improves respiratory reserve and decreases hyperactivity of accessory breathing muscles during respiratory excursions: A randomized controlled trial. Acta Bioeng. Biomech..

[37] Keter D.L., Bialosky J.E., Brochetti K., Courtney C.A., Funabashi M., Karas S., Learman K., Cook C.E. (2025). The mechanisms of manual therapy: A living review of systematic, narrative, and scoping reviews. PLoS ONE.

[38] McLaughlin L., Goldsmith C.H., Coleman K. (2011). Breathing evaluation and retraining as an adjunct to manual therapy. Man. Ther..

[39] Busch V., Magerl W., Kern U., Haas J., Hajak G., Eichhammer P. (2012). The effect of deep and slow breathing on pain perception, autonomic activity, and mood processing—An experimental study. Pain Med..

[40] Dimitriadis Z., Kapreli E., Strimpakos N., Oldham J. (2015). Do psychological states associate with pain and disability in chronic neck pain patients?. J. Back Musculoskelet. Rehabil..

