# Supplementary material for: The Effectiveness of Cervical Spine and Diaphragm Manual Therapy Combined with Breathing Re-Education Exercises on Musculoskeletal, Respiratory and Psychophysiological Outcomes in Patients with Non-Specific Chronic Neck Pain: A Randomized Controlled Trial
Source: J Clin Med. 2026 May 31;15(11):4266. doi: 10.3390/jcm15114266 (PMC13257620; doi:10.3390/jcm15114266)
Supplement: Supplementary file 1 [file jcm-15-04266-s001.zip › Supplementary Material S1.pdf]

Supplementary Material – Outcome Measures and Adverse Events Monitoring

I. MUSCULOSKELETAL-BASED OUTCOMES

Primary Outcomes Measures

Pain intensity numeric rating scale (PI-NRS)

Pain intensity was assessed using a pain intensity numeric rating scale (PI-NRS), with participants asked to rate their average pain intensity over the last week on a scale from 0 to 10. The left end of the line was labeled "0 - no pain," while the right end was labeled "10 - worst pain imaginable" [1]. A minimum clinically important difference (MCID) of 1.5 points with a minimum detectable change (MDC) of 2.5 points in the NRS has been previously reported in 107 patients with mechanical neck pain without arm symptoms [2].

Neck Disability Index (NDI)

The Neck Disability Index (NDI) is a valid, reliable, and responsive tool for detecting changes in disability-related daily activities before and after therapeutic interventions in patients with neck pain, also validated for Greek primary health care settings [3]. The NDI has 10 questions, pertaining to the ease of task completion, pain intensity, and the presence of headaches. The score for each question varies from 0 (no pain / functional limitation) to 5 (extreme pain / maximum limitation). The total score ranges from 0 (indicating no disability) to 50 (indicating complete disability), and if doubled, the scale represents a percentage. The Neck Disability Index percentage (NDI%) was used in this study. An MCID of 11 points with a minimum detectable change (MDC) of 13.8 points in the NDI % 0-100 scale has been previously reported [2].

Secondary Outcomes Measures

As patients with NSCNP present with a multifaceted array of signs and symptoms, we selected clinical outcomes that could capture most aspects of their condition.

Tampa Scale for Kinesiophobia (TSK)

Kinesiophobia is the fear of movement and/or re-injury frequently assessed by the TSK scale, a valid and reliable instrument to assess patients' perceptions of fear of re-injury due to physical activity. The TSK has also been validated in Greek [4]. The TSK consists of 17 questions, each with four possible responses ranging from 1 (strongly disagree) to 4 (strongly agree). The total score is calculated after reversing the scores for questions 4, 8, 12, and 16. The final score ranges from 17 to 68. A higher score indicates a greater degree of fear experienced by the patient, with a score of 37

or less suggesting a reduced fear of movement or injury [5]. An MCID of 9.5 points in the 17-item TSK has been previously reported in patients with chronic neck pain [6].

Hospital Anxiety and Depression Scale (HADS)

The Hospital Anxiety and Depression Scale (HADS) is a valid, reliable, and user-friendly questionnaire for assessing anxiety and depression also available in Greek [7]. The scale is self-administered and consists of 14 items in total, with half of the items relating to anxiety (HADS-A) and the remaining assessing depression (HADS-D). Each item has 4 possible responses (0–3), with scores for each subscale ranging from 0 to 21. An MCID of 1.17 to 2.13 for HADS-A, and 1.48 to 2.54 for HADS-D has been reported in patients with chronic pain [8].

II. <u>PSYCHO-PHYSIOLOGICAL OUTCOME</u>

Nijmegen Questionnaire (NQ)

The Nijmegen Questionnaire (NQ) [9] is a self-report instrument comprising 16 items. These items are rated on a five-point Likert scale: 0 (never), 1 (rarely), 2 (sometimes), 3 (often), and 4 (very often). A score of ≥19 [10], or ≥20 suggests the presence of hyperventilation syndrome in the general population [11]. The 16 NQ items address respiratory, cardiovascular, neurological, gastrointestinal, and psychological symptoms [9], which are common in both asthma and anxiety [12]. The NQ has demonstrated satisfactory test-retest reliability (r=0.70) and high sensitivity and specificity (91% and 95% respectively) in a healthy population with diagnosed hyperventilation syndrome [9] and NQ's measurement properties are strong enough to support its use in a clinical setting for diaphragmatic breathing [13]. The questionnaire has also been translated and validated for the Greek population with asthma [14]. There have been no MCID values reported for the NQ [15].

III. <u>RESPIRATORY OUTCOMES</u>

Hi- Lo test

The Hi-Lo test is a specific assessment tool used to detect dysfunctional breathing [16]. This manual assessment helps determine if a patient exhibits diaphragmatic/normal breathing or an abnormal breathing pattern. The test is performed with the patient seated and the examiner standing or sitting beside them, slightly diagonally in front. Then, the examiner placed one hand on the patient's sternum and one hand on their upper abdomen. While the patient was quietly breathing, the examiner should have determined whether thoracic or abdominal motion was dominant during breathing and to what extent this was so. Also, to check for paradoxical breathing by seeing if the abdomen moved in a direction opposite to the thorax during breathing. The examiner then ticked a box indicating if they considered the breathing pattern to be predominantly abdominal or thoracic.

Assessment of paradoxical breathing was also recorded by ticking a box [16,17]. The reliability and validity of the Hi-Lo test have been studied, with Courtney et al. 2009 [16], specifically supporting the validity and ease of use of the Hi-Lo test compared to the MARM assessment scale.

Breath Holding Time (BHT) test

The Breath Holding Time (BHT) test is a specific assessment used to detect hyperventilation and disordered breathing patterns [18]. Participants were asked to sit relaxed and breathe calmly and normally. Following an exhalation, patients were instructed to pinch their nose and hold their breath. Measurements were taken using a Huawei P30 ELE-L29 mobile phone stopwatch, which measures time to 0.01 seconds. Each measurement was performed three times, and the average of these measurements was used. The instruction given was: "Hold your breath until you feel the need to inhale again." Failing to hold one's breath for more than 30 seconds is considered a positive diagnostic indicator of chronic hyperventilation [17,19].

Single Breath Count (SBC) test

The Single Breath Count (SBC) test, a specific assessment for detecting dysfunctional breathing, was performed in accordance with the study by Bartfield et al (1994)[20]. Each participant was instructed to take a maximal inspiration (Total Lung Capacity - TLC) and then begin counting aloud at a normal voice volume, following a metronome on a mobile phone (providing both visual and auditory stimuli) at a rate of two numbers per second, until they felt the need to inhale.

According to Kukulka et al. (2020) [21], values greater than 25 seconds (i.e., 50 counts) indicate a normal cardiopulmonary reserve; values between 15 and 25 seconds indicate a limited reserve, and values less than 15 seconds indicate that the cardiopulmonary reserve is considered very poor.

Capnography - End Tidal CO_2 and Respiratory Rate (RR)

A capnograph (Nonin Medical Inc, LifeSense® II) was used to measure the carbon dioxide (CO2) present in each exhalation, known as End-Tidal Carbon Dioxide (ETCO2). CO2 is a byproduct of cellular metabolism that enters the lungs from venous circulation and is expelled during exhalation. ETCO2 values of less 35 mmHg are associated with hypocapnia [17]. The capnograph displays values numerically and graphically and calibration is essential because results are affected by environmental humidity and temperature, barometric pressure, and altitude. We collected data for ten minutes while the participant was in a seated position and calm, with their mobile phone switched off and they were advised not to speak or be distracted in any way [18].

The capnograph device also allows for the calculation of Respiratory Rate (RR) per minute. The depth and frequency of breathing represent the amount of oxygen (O_2)

and carbon dioxide (CO_2) exchanged during respiration. A higher-than-normal respiratory rate during hyperventilation leads to respiratory alkalosis due to a lack of CO_2 [17].

Chest Wall Expansion (CWE)

Chest Wall Expansion (CWE) was measured using a flexible tape measure (0-100 cm) in standing. The tape was positioned at two levels: first at the axillary level and then at the xiphoid process. Measurements were taken at both maximal inspiration and maximal expiration. The reliability of this measurement method has been previously verified [22].

Adverse events

A questionnaire for assessing potentially undesirable events that might have occurred during, at the end of, or after treatment was used. Each patient completed the questionnaire in the waiting area before their scheduled session considering the type of adverse event (fatigue, muscle discomfort, stiffness, pain increase, nausea, headache, dizziness, or other), its intensity, and its duration. After its completion, the treating physiotherapist checked the questionnaire for completeness and accuracy of responses. If a patient had not shown up for their appointment, the therapist was required to contact them and reschedule [23].

REFERENCES

1. Euasobhon, P.; Atisook, R.; Bumrungchatudom, K.; Zinboonyahgoon, N.; Saisavoey, N.; Jensen, M.P. Reliability and responsivity of pain intensity scales in individuals with chronic pain. *Pain* **2022**, *163*, e1184-e1191, doi:10.1097/j.pain.0000000000002692.
2. Young, I.; Dunning, J.; Butts, R.; Mourad, F.; Cleland, J. Reliability, construct validity, and responsiveness of the neck disability index and numeric pain rating scale in patients with mechanical neck pain without upper extremity symptoms. *Physiother Theory Pract* **2019**, *35*, 1328-1335, doi:10.1080/09593985.2018.1471763.
3. Trouli, M.N.; Vernon, H.T.; Kakavelakis, K.N.; Antonopoulou, M.D.; Paganas, A.N.; Lionis, C.D. Translation of the Neck Disability Index and validation of the Greek version in a sample of neck pain patients. *BMC Musculoskelet Disord* **2008**, *9*, 106, doi:10.1186/1471-2474-9-106.
4. Georgoudis, G.; Raptis, K.; Koutserimpas, C. Cognitive Assessment of Musculoskeletal Pain: Validity and Reliability of the Greek Version of the Tampa Scale of Kinesiophobia in Patients Suffering from Chronic Low Back Pain. *Maedica (Bucur)* **2022**, *17*, 826-832, doi:10.26574/maedica.2022.17.4.826.

5. Dupuis, F.; Cherif, A.; Batcho, C.; Massé-Alarie, H.; Roy, J.S. The Tampa Scale of Kinesiophobia: A Systematic Review of Its Psychometric Properties in People With Musculoskeletal Pain. *Clin J Pain* **2023**, *39*, 236-247, doi:10.1097/ajp.0000000000001104.
6. Saadat, M.; Salamat, S.; Mostafaee, N.; Soleimani, F.; Rouintan, Z.; Amin, M. To evaluate responsiveness and minimal important change (MIC) for the Persian versions of FABQ, TSK, and PCS. *Eur Spine J* **2023**, *32*, 3023-3029, doi:10.1007/s00586-023-07835-w.
7. Michopoulos, I.; Douzenis, A.; Kalkavoura, C.; Christodoulou, C.; Michalopoulou, P.; Kalemi, G.; Fineti, K.; Patapis, P.; Protopapas, K.; Lykouras, L. Hospital Anxiety and Depression Scale (HADS): validation in a Greek general hospital sample. *Ann Gen Psychiatry* **2008**, *7*, 4, doi:10.1186/1744-859x-7-4.
8. Grönkvist, R.; Vixner, L.; Äng, B.; Grimby-Ekman, A. Measurement Error, Minimal Detectable Change, and Minimal Clinically Important Difference of the Short Form-36 Health Survey, Hospital Anxiety and Depression Scale, and Pain Numeric Rating Scale in Patients With Chronic Pain. *J Pain* **2024**, *25*, 104559, doi:10.1016/j.jpain.2024.104559.
9. van Dixhoorn, J.; Duivenvoorden, H.J. Efficacy of Nijmegen Questionnaire in recognition of the hyperventilation syndrome. *J Psychosom Res* **1985**, *29*, 199-206, doi:10.1016/0022-3999(85)90042-x.
10. van Dixhoorn, J.; Folgering, H. The Nijmegen Questionnaire and dysfunctional breathing. *ERJ Open Res* **2015**, *1*, doi:10.1183/23120541.00001-2015.
11. Azizmohammad, L.; Masaebi, F.; Abedi, M.; Mohseni, N.; Fakharian, A. The Optimal Cut-off Score of the Nijmegen Questionnaire for Diagnosing Hyperventilation Syndrome Using a Bayesian Model in the Absence of a Gold Standard. *Galen Med J* **2020**, *9*, e1738, doi:10.31661/gmj.v9i0.1738.
12. Meuret, A.E.; Ritz, T. Hyperventilation in panic disorder and asthma: empirical evidence and clinical strategies. *Int J Psychophysiol* **2010**, *78*, 68-79, doi:10.1016/j.ijpsycho.2010.05.006.
13. Mohan, V.; Rathinam, C.; Yates, D.; Paungmali, A.; Boos, C. Validity and reliability of outcome measures to assess dysfunctional breathing: a systematic review. *BMJ Open Respir Res* **2024**, *11*, doi:10.1136/bmjresp-2023-001884.
14. Grammatopoulou, E.P.; Skordilis, E.K.; Georgoudis, G.; Haniotou, A.; Evangelodimou, A.; Fildissis, G.; Katsoulas, T.; Kalagiakos, P. Hyperventilation in asthma: a validation study of the Nijmegen Questionnaire--NQ. *J Asthma* **2014**, *51*, 839-846, doi:10.3109/02770903.2014.922190.
15. Li Ogilvie, V.; Kersten, P. A critical review of the psychometric properties of the Nijmegen Questionnaire for hyperventilation syndrome. *New Zealand Journal of Physiotherapy* **2015**, *43*, 3–10, doi:10.15619/NZJP/43.1.01.
16. Courtney, R.; Cohen, M.; Reece, J. Comparison of the Manual Assessment of Respiratory Motion (MARM) and the Hi Lo Breathing Assessment in determining a simulated breathing pattern. *International Journal of Osteopathic Medicine* **2009**, *12*, 86-91, doi:10.1016/j.ijosm.2008.10.002.
17. Kiesel, K.; Rhodes, T.; Mueller, J.; Waninger, A.; Butler, R. Development of a screening protocol to identify individuals with dysfunctional breathing. *Int J Sports Phys Ther* **2017**, *12*, 774-786.

18. Courtney, R.; Greenwood, K.M.; Cohen, M. Relationships between measures of dysfunctional breathing in a population with concerns about their breathing. *J Bodyw Mov Ther* **2011**, *15*, 24-34, doi:10.1016/j.jbmt.2010.06.004.
19. Chaitow, L. Breathing pattern disorders, motor control, and low back pain. *Journal of Osteopathic Medicine* **2004**, *7*, 33-40, doi:https://doi.org/10.1016/S1443-8461(04)80007-8.
20. Bartfield, J.M.; Ushkow, B.S.; Rosen, J.M.; Dylong, K. Single breath counting in the assessment of pulmonary function. *Ann Emerg Med* **1994**, *24*, 256-259, doi:10.1016/s0196-0644(94)70138-5.
21. Kukulka, K.; Gummi, R.R.; Govindarajan, R. A telephonic single breath count test for screening of exacerbations of myasthenia gravis: A pilot study. *Muscle Nerve* **2020**, *62*, 258-261, doi:10.1002/mus.26987.
22. Mohan, V.; Dzulkifli, N.; Justine, M.; Haron, R.; Joseph H, L.; Rathinam, C. Intrarater Reliability of Chest Expansion using Cloth Tape Measure Technique. *Bangladesh Journal of Medical Science* **2012**, *11*, 307-311, doi:10.3329/bjms.v11i4.12602.
23. Paanalahti, K.; Holm, L.W.; Nordin, M.; Asker, M.; Lyander, J.; Skillgate, E. Adverse events after manual therapy among patients seeking care for neck and/or back pain: a randomized controlled trial. *BMC Musculoskelet Disord* **2014**, *15*, 77, doi:10.1186/1471-2474-15-77.
